# A Dual-Cuboid Constraint Method with Marching Cubes-Based Contour Extraction for Verticality Inspection of Square Tower Structures

**DOI:** 10.3390/s26154918

**Published:** 2026-08-04

**Authors:** Mingduan Zhou, Zihan Zhou, Yuhan Qin, Guanxiu Wu, Qiao Song, Shiqi Lin, Lu Qin, Peng Yan, Shufa Li

**Affiliations:** School of Geomatics and Urban Spatial Informatics, Beijing University of Civil Engineering and Architecture, Beijing 102616, China

**Keywords:** LiDAR point cloud, square tower structures, verticality inspection, constraints, central axis fitting, a dual-cuboid constraint frame

## Abstract

Verticality inspection of square tower structures is of great significance for ensuring structural operational safety, extending service life, and optimizing maintenance strategies. However, conventional verticality inspection methods still exhibit deficiencies in point cloud noise suppression, removal of internal redundant points, and stable geometric feature extraction, which often lead to fluctuations in axis fitting results and fail to meet the requirements of non-contact, high-precision, and holistic perception-based verticality inspection for square tower structures. To address the above issues, this paper proposes a Dual-Cuboid Constraint Method with Marching Cubes-Based Contour Extraction for Verticality Inspection of Square Tower Structures. First, raw point cloud data of the surface of square tower structures were acquired through terrestrial LiDAR multi-station scanning. After preprocessing, including point cloud registration, coordinate system unification, redundancy removal, and denoising, high-precision effective point cloud data were obtained in a unified station-centered spatial coordinate system. Subsequently, the initial body center point coordinates and principal axis directions of the standard segment point clouds were determined using the principal axis projection method. Based on these results, a dual-cuboid constraint frame was constructed, and the shell point clouds of the standard segments were extracted through outer-frame enclosure and inner-frame exclusion. The principal axis projection method was then applied again to calculate the body center point coordinates of the shell point cloud for each standard segment. The central axis of the body center point set was subsequently fitted using the least squares method to determine the unit direction vector of the structural central axis. Finally, within the station-centered spatial coordinate system, vector operations were performed between the unit direction vector and the x-axis and z-axis, respectively, to calculate the tilt attitude parameters of the square tower structure, including the azimuth angle of inclination, the inclination angle, and the verticality. The detection results obtained from different schemes were then comparatively evaluated using the relative error metric. Field validation was conducted on a tower crane at a construction site in Beijing. Four dual-cuboid constraint frame schemes with dimensional errors of 20 mm, 40 mm, 60 mm, and 80 mm were designed. Furthermore, based on the experimental process of the method presented in this paper, an unconstrained solution was established as a comparison experiment, and it was compared with the two reference experiments based on the Marching Square algorithm and the RANSAC algorithm. The results indicated that the tower-body verticality values obtained using the four dual-cuboid constraint frame schemes with different dimensional errors were 3.48‰, 3.63‰, 3.60‰, and 3.82‰, respectively, with a mean value of 3.63‰ and a range of only 0.34‰. The verticality results obtained from the four constrained schemes were generally consistent and showed good agreement with those obtained from 3.03‰ and 3.14‰, and reasonable proximity to the result obtained from 2.82‰, respectively. The results further suggest that the proposed dual-cuboid constraint frame contributes to improving the stability of point cloud feature extraction and central axis fitting, and yields generally consistent detection results across different dimensional error parameters, indicating that the method exhibits robustness to variations in the constraint-frame dimensions within the tested parameter ranges.

## 1. Introduction

With the rapid advancement of urbanization, the number and scale of square tower structures in urban environments have increased substantially, including high-rise buildings and various large, slender, and square tower structures [1,2,3]. Compared with conventional engineering structures, square tower structures are typically characterized by significant height, high slenderness ratios, and complex loading conditions. Under the combined effects of wind loads, seismic actions, and environmental variations, these structures are susceptible to global geometric deformation [4,5,6,7,8]. During long-term service, these structures may be influenced by material degradation and structural configurations, which may cause the structural axis to shift or tilt [9,10]. Axis deviation or inclination may reduce the overall structural stability of the square tower structures; in severe cases, it may cause structural cracking, instability, and even overturning, resulting in safety accidents. Therefore, achieving high-precision, non-contact, and holistic verticality inspection is of great significance for the safety assessment, risk warning, and life extension management of square tower structures.

Currently, Terrestrial Laser Scanning (TLS) technology, due to its non-contact nature and high-precision, high-density data acquisition capabilities, is gradually being widely applied in deformation inspection and has demonstrated considerable advantages in a wide range of engineering applications. Tao F [11] addressed the difficulties of point deployment and the lack of global representation in traditional slope monitoring. Based on multi-temporal TLS point cloud data, through point cloud registration, denoising, and three-dimensional modeling, deviation analysis was further combined to achieve high-precision quantitative evaluation of the overall deformation of large-scale slopes. The results showed that the deviations of most point clouds were controlled within ±20 mm. Wang R [12] obtained high-density point cloud data through multi-station deployment and combined cross-sectional slicing with central-axis node offset analysis methods to achieve precise identification of spatial deflection, inclination, and local deformation of steel structure components. Xian Z et al. [13] utilized the advantages of TLS point cloud’s high-density and non-contact acquisition of three-dimensional information, proposing a block ICP displacement inversion method based on local sub-block rigid registration and two-stage quality control to extract continuous and clearly defined three-dimensional displacement fields. Ning X et al. [14] constructed an ICP-based multi-period point cloud registration model using TLS, directly establishing correspondences between the same spatial points and achieving three-dimensional deformation calculation with more realistic physical significance. The accuracy was significantly higher than that of traditional methods based on nearest-distance computation. Pexman K et al. [15] realized high-precision estimation of surface normals of aerospace components through TLS point clouds, verifying its application potential in precision manufacturing. Richard N et al. [16] combined machine learning methods to predict pavement macrotexture parameters from TLS data. Chen Z et al. [17] proposed a TLS station-planning method based on three-dimensional models, improving the efficiency and reliability of large-scale building façade data acquisition. Tysiac P et al. [18] integrated TLS with unmanned aerial vehicle (UAV) image measurement to achieve high-precision three-dimensional reconstruction and disease assessment of historical buildings. Jo Y et al. [19] constructed a three-dimensional model of cultural heritage through the fusion of TLS and UAV data, verifying the advantages of TLS in geometric accuracy. Ling X [20] analyzed the factors affecting TLS measurement accuracy, providing a theoretical basis for engineering applications. Pawlowicz J et al. [21] acquired high-density point cloud data of buildings using terrestrial laser scanning, combined with point cloud processing and geometric analysis, to achieve comprehensive assessment of the geometric morphology of building structures, component deformation, and local damage. These studies collectively demonstrate the superior performance and excellent overall expression and detailed depiction capabilities of TLS technology in engineering practice from multiple aspects such as data acquisition, feature extraction, and deformation analysis, providing a reliable data basis for geometric morphology analysis of complex structures.

Owing to the advantages of TLS technology [22,23,24,25], numerous studies have applied TLS technology to structural verticality inspection and demonstrated its effectiveness in engineering applications [26,27]. Kaszowska O et al. [28] introduced TLS technology in industrial chimney monitoring, obtaining high-density point cloud data of the chimney to construct a three-dimensional model, conducting layered analysis of different height sections, extracting the center positions of each section, and achieving the characterization of the spatial variation characteristics and verticality changes in the chimney axis. Głowacki T et al. [29] used TLS technology to conduct high-precision three-dimensional measurement of cooling towers; through point cloud reconstruction of the actual geometric shape and comparison analysis with the design model, they obtained the overall shape deviation distribution of the tower body and achieved quantitative assessment of the deformation characteristics of tall structures. Zhou M et al. [30] optimized the contour extraction and center axis fitting process based on the Marching Square algorithm, achieving precise evaluation of the overall verticality of slender tower structures. Kregar K et al. [31] combined the point cloud data obtained from TLS, using the least squares method to fit the cylindrical model of the chimney, then extracting the structural center axis and calculating its inclination angle, thus achieving precise calculation of the verticality of tall structures. Daliga K et al. [32] determined the axis of a stainless steel chimney based on laser scanning data, analyzing the geometric parameters of the sections and the center offset to evaluate the degree of axis deviation. Zhou M et al. [33] proposed a non-contact inclined detection method based on an improved algorithm and applied it to the verticality detection of tower cranes [34]. Pleterski Ž et al. [35] based on TLS data, using the RANSAC algorithm combined with least squares circle/ellipse fitting, achieved efficient evaluation of chimney axis curvature and non-verticality. Kitratporn N et al. [36] took the Pathein suspension bridge in Myanmar as the research object and successfully measured three deformation behaviors—tower column inclination, cable inclination, and truss deflection—using TLS point cloud data. This demonstrated the advantages of TLS in measuring inaccessible areas. Beshr A et al. [37] projected the surface points of four cooling towers in Egypt onto a vertical plane. Using TLS observation values and least squares fitting, they determined the tower wall equation, calculated the axis inclination, and identified minor deformations. Popović J et al. [38] proposed a method for quantifying the inclination trend based on TLS feature point extraction for tall structures. They evaluated the evolution of the structural inclination by analyzing the changes in the central axis from multiple scans. Makuch M et al. [39] employed TLS technology. By extracting the point cloud of the horizontal section and fitting the center of the circle, they discovered that the cooling tower had a permanent inclination toward the northeast, achieving the assessment of the inclination and geometric defects of a large hyperbolic surface cooling tower. Zhou M et al. [40] utilized LiDAR point clouds and adaptive slicing strategies to achieve high-precision verticality monitoring of ultra-high chimneys. All the above verticality detection studies achieved good detection results, but most existing studies did not introduce effective geometric constraints during point cloud processing and feature extraction. Consequently, the stability of geometric feature extraction remains insufficient, which makes the reliability of the verticality inspection parameter calculation prone to being affected due to the instability of feature extraction. However, the standard segments of open-frame square tower structures contain external chords, diagonal braces, connection plates, internal structural members, and locally distributed interference points. These points may occur simultaneously within the point cloud of the same standard segment. When section centers or structural axes are calculated directly from unconstrained point clouds, geometric feature extraction may be affected by internal members, uneven point distributions, occlusion, and local noise. Therefore, an explicit mechanism is required to determine which points should participate in subsequent contour extraction and body center point calculation.

During point cloud processing, the appropriate incorporation of geometric constraints can effectively suppress noise interference, reduce the influence of outliers, improve the stability of point cloud registration, denoising, segmentation, and geometric feature extraction, and enhance the representation of the three-dimensional geometric characteristics of complex structures. Consequently, extensive research has been conducted in recent years on the application of geometric constraints in point cloud processing, leading to significant advances. Zheng J et al. [41] introduced closure conditions and external geometric constraints to achieve high-precision global registration of multi-station point clouds for high-speed railway bridges, thereby improving the accuracy and robustness of long-distance point cloud registration. Kang C et al. [42] proposed a point cloud registration method based on geometric constraints and transformation matrix evaluation, in which rigid constraints and salient-point distance constraints were employed to identify high-quality corresponding points, thereby improving both registration accuracy and computational efficiency. David Y et al. [43] developed a point cloud denoising model by integrating color information, confidence estimation, and geometric constraints, effectively suppressing noise in satellite photogrammetric point clouds while enhancing the preservation of geometric details. Hibiya H et al. [44] reconstructed the actual deformation state in structural finite element analysis by establishing deformation surface constraints and normal constraints from terrestrial LiDAR point clouds, thereby improving the fidelity of structural deformation analysis. Zhang Y et al. [45] proposed a Sight View Constraint method that improved the robustness of point cloud registration in low-overlap scenarios by eliminating incorrect transformation relationships. Gou F et al. [46] achieved refined registration of planar tunnel point clouds by jointly incorporating ground constraints, distance constraints, and normal constraints, thereby improving the registration accuracy and stability in complex environments. Lv Y et al. [47] established a point cloud registration model for steel tubular arch bridge segments based on local geometric constraints and combined circular cross-sectional constraints with the Coherent Point Drift (CPD) algorithm to achieve high-precision registration of complex steel structures. Liu H et al. [48] improved the accuracy of mobile laser scanning point clouds in subway environments through target constraints, dynamic calibration, and multi-source data fusion, providing a reliable solution for high-precision point cloud acquisition in underground environments. Ren Z et al. [49] proposed a dynamic point cloud extraction method based on global geometric constraints by constructing a polar-coordinate geometric constraint model, enabling accurate extraction of point clouds in complex roadway structures while significantly improving processing robustness. Yu S et al. [50] proposed a street-tree point cloud segmentation method integrating multiple constraints with shortest-path optimization, in which structural constraints and trunk-axis analysis were employed to achieve accurate segmentation of individual trees in complex urban environments. Ran X et al. [51] achieved high-precision planar segmentation of sensor-acquired point clouds by exploiting supervoxel boundary adjacency, normal consistency, and projected line-fitting constraints, thereby improving planar recognition accuracy in complex geometric scenes. Yang J et al. [52] proposed a label-constrained building roof detection method that integrated geometric and spectral information to achieve high-precision identification of building roof regions from airborne LiDAR data. Li Y et al. [53] developed an unsupervised domain adaptation framework based on point cloud structural constraints, in which three-dimensional contour point cloud constraints were introduced to improve cross-domain contour segmentation accuracy in medical images. Li M et al. [54] introduced consistency constraints and a position-guided mechanism for weakly supervised semantic segmentation of large-scale indoor point clouds, thereby improving both model generalization and segmentation accuracy. Weng H et al. [55] proposed a Gaussian Splatting reconstruction framework that integrates LiDAR point clouds with spatial constraints, in which local surface geometric constraints were employed to achieve high-fidelity three-dimensional reconstruction while reducing model complexity. Zheng D et al. [56] proposed a building point cloud registration algorithm based on geometric feature constraints, in which point, line, and plane geometric feature constraints were incorporated into the registration model, thereby improving the accuracy and stability of building point cloud registration. These studies have collectively demonstrated that geometric constraints can effectively improve point cloud processing accuracy, enhance model stability, and strengthen the geometric representation of complex structures in tasks such as point cloud registration, denoising, target segmentation, three-dimensional reconstruction, and geometric feature extraction. The geometric constraint methods discussed above were developed primarily for intermediate processing tasks, such as point cloud registration, segmentation, and reconstruction. Their constraints are imposed at specific stages of point cloud processing and generally act on point-to-point spatial relationships or the overall geometric consistency of the point cloud, rather than systematically constraining the shell geometry of structural standard segments. To the best of the authors’ knowledge, no previous study has systematically established an inner–outer nested spatial-range constraint framework for the standard segment point clouds of square tower structures to achieve the integrated processing of stable shell point cloud extraction and central axis fitting.

To address these limitations, this paper proposes a Dual-Cuboid Constraint Method with Marching Cubes-Based Contour Extraction for Verticality Inspection of Square Tower Structures. The main contributions of this paper are as follows: (1) Construction of a dual-cuboid constraint frame: To address the discrete spatial distribution of point cloud data and the interference caused by noise, an inner–outer nested dual-cuboid constraint frame is constructed. By constraining the spatial extent of the point cloud and its geometric relationships, effective structural points are stably extracted and represented, thereby improving the accuracy and stability of point cloud processing. (2) Three-dimensional contour feature extraction: Under the dual-cuboid constraint frame, three-dimensional contour lines of square tower structures are constructed based on different size parameters of the dual-cuboid constraint frame, enabling a complete representation of the external geometric characteristics of the structures. (3) Global three-dimensional central axis fitting: Based on the body center point coordinates, the central axis is fitted to achieve unified modeling and parameter estimation of the three-dimensional global central axis. (4) Field validation and performance evaluation: Field experiments are conducted using real engineering point cloud data, and comparative analyses with conventional methods are performed to verify the accuracy and stability of the proposed method.

The remainder of this paper is organized as follows: Section 2 introduces the research methods and materials; Section 3 elaborates on the experimental details; Section 4 presents the results of the verticality inspection; Section 5 analyzes and discusses the experimental results; Section 6 concludes the research and makes prospects.

## 2. Methods and Materials

### 2.1. Methods

The framework of a Dual-Cuboid Constraint Method with Marching Cubes-Based Contour Extraction for Verticality Inspection of Square Tower Structures is described as follows:

First, high-precision point cloud data of the square tower structure are acquired. Terrestrial LiDAR multi-station scanning is employed to obtain the raw point cloud data of the square tower structure. Through preprocessing such as point cloud registration between stations, coordinate unification, redundancy removal, and denoising, high-precision effective point cloud data in a unified station-centered spatial coordinate system are obtained.

Secondly, the dual-cuboid constraint frame is constructed and the central axis direction is extracted. To address the internal redundant points and external noise points contained in the standard segment point clouds of square tower structures, the initial body center point coordinates and principal axis directions of the standard segment point clouds are first calculated using the principal axis projection method. The body center point of each standard segment point cloud is then taken as the alignment reference for constructing a dual-cuboid geometric constraint frame, thereby enabling spatial constraint-based filtering of the standard segment point clouds and extracting the shell point cloud that represents the external contour of each standard segment. Subsequently, the principal axis projection method is applied again to the shell point cloud to calculate the body center point coordinates, thereby forming a set of spatial body center points for central axis fitting. Finally, the spatial line representing the central axis of the square tower structure is fitted using the least squares method, and the unit direction vector of the central axis is determined.

**Figure 1:**
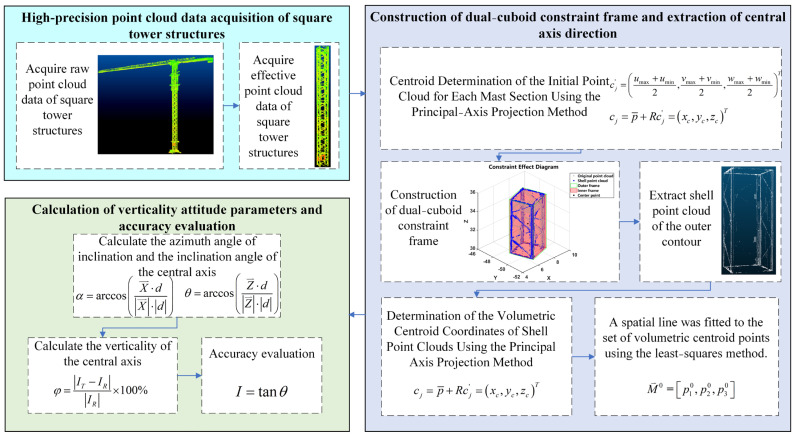
Flowchart of the proposed method.

Finally, the verticality-related attitude parameters are calculated, and the corresponding accuracy is evaluated. In the terrestrial LiDAR station-centered spatial coordinate system, vector operations are respectively performed between the unit direction vector of the central axis and the x-axis unit vector [1,0,0] as well as the z-axis unit vector [0,0,1] to determine the verticality-related attitude parameters of the central axis of the square tower structure, including the azimuth angle of inclination, the inclination angle, and the verticality of the tower body. The corresponding workflow of the dual-cuboid constraint method for inspecting the verticality of square tower structures using the Marching Cubes algorithm is illustrated in Figure 1.

#### 2.1.1. High-Precision Point Cloud Data Acquisition of Square Tower Structures

Acquisition of raw point cloud data of square tower structures. At suitable locations with unobstructed views and stable ground conditions around the square tower structure, several scanning stations are freely set up at different orientations and distances to perform full-sphere terrestrial LiDAR measurements, and the raw point cloud data of the square tower structure are acquired by scanning.

Acquisition of effective point cloud data of square tower structures. For the raw point cloud data acquired from each scanning station, point cloud registration and coordinate transformation between adjacent stations are carried out using targets or homologous points [57,58], followed by necessary preprocessing such as redundancy removal and denoising. As a result, effective high-precision point cloud data of the square tower structure in the same station-centered spatial coordinate system are obtained. The station-centered coordinate system is a local relative coordinate system, in which the origin, O, is defined as the center of the terrestrial LiDAR scanner at the first scanning station. The z-axis is aligned with the vertical direction, while the x-axis is defined by the initial orientation of the scanner. The x-axis and y-axis are orthogonal within the horizontal plane, forming a north–east–up (NEU) Cartesian coordinate system, denoted as *o-xyz*, with the first scanning station serving as the reference. The point cloud data in the station-centered spatial coordinate system *o-xyz* can be expressed as Equation (1):

(1) P=(x,y,z)=(S⋅cosθ⋅cosα,S⋅cosθ⋅sinα,S⋅sinθ)

where P is the point cloud data; S is the measured distance from the LiDAR laser beam to the surface of the square tower structure; α is the horizontal deflection angle of the LiDAR laser beam; θ is the vertical deflection angle of the LiDAR laser beam.

#### 2.1.2. Standard Segment Partitioning and Body Center Point Calculation of Square Tower Structures

1. Standard segment partitioning of square tower structures. Within the station-centered spatial coordinate system *o-xyz*, the preprocessed surface point cloud model of the square tower structure is transversely partitioned into cuboid point clouds along the z-axis to obtain the standard segment point cloud. Based on the resulting standard segment point cloud, the spatial coordinates of all points contained within each standard segment are determined. Accordingly, the point cloud coordinate set of the standard segment can be expressed as Equation (2):
  (2) $$P_{j}=\left\{p_{i}=(x_{i},y_{i},z_{i})\right\}$$
  where $P_{j}$ denotes the point cloud coordinate set of the j-th standard segment $\left(j=1,2,\cdots ,m\right)$, where m is the total number of standard segments. $p_{i}$ represents the i-th three-dimensional point in the standard segment $\left(i=1,2,\cdots ,n_{j}\right)$, and $n_{j}$ denotes the number of point cloud points contained in the j-th standard segment.
2. Body center point calculation of the standard segments of square tower structures. To accurately determine the spatial body center point of each standard segment, principal component analysis (PCA) is employed to establish a local coordinate system for the standard segment, in which the three principal axes are aligned with the length, width, and height directions of the standard segment, respectively. An oriented bounding box (OBB) is then constructed based on the principal axis projections, and its geometric center is calculated as the body center point of the standard segment. The specific procedure is as follows.
  Step 1: The centroid of the standard segment point cloud of the square tower structure is calculated to eliminate the influence of coordinate translation, as expressed in Equation (3):
  (3) $${\bar{p}}_{j}=\left(\frac{1}{n_{j}}\sum_{i=1}^{n_{j}}x_{i},\frac{1}{n_{j}}\sum_{i=1}^{n_{j}}y_{i},\frac{1}{n_{j}}\sum_{i=1}^{n_{j}}z_{i}\right)=\left(x_{j},y_{j},z_{j}\right)$$
  where ${\bar{p}}_{j}=(x_{j,}y_{j},z_{j})$ denotes the centroid of the j-th standard segment.
  Step 2: The point cloud is centralized to construct the covariance matrix, as expressed in Equation (4):
  (4) $$C_{j}=\frac{1}{n_{j}}\sum_{i=1}^{n_{j}}\left(p_{i}-\bar{p}\right){\left(p_{i}-\bar{p}\right)}^{T}$$
  where $C_{j}$ denotes the covariance matrix of the j-th standard segment, and $p_{i}-\bar{p}$ represents the centralized point cloud.
  Step 3: Eigenvalue decomposition is performed on the covariance matrix to construct the rotation matrix, as expressed in Equation (5):
  (5) $$\left\{\begin{array}{l} C_{j}e_{j}=\lambda_{j}e_{j}\\ R=\left[e_{1},e_{2},e_{3}\right] \end{array}\right.$$
  where $\lambda_{j}$ denotes the eigenvalues, and $e_{j}$ represents the corresponding unit eigenvectors. R is the rotation matrix composed of the three eigenvectors arranged in descending order according to their corresponding eigenvalues.
  Step 4: The point cloud is projected onto the principal axis coordinate system using the rotation matrix, and the projected coordinates are expressed as Equation (6):
  (6) $$p_{i}^{\prime }=R^{T}\left(p_{i}-\bar{p}\right)={\left(u_{i},v_{i},w_{i}\right)}^{T}$$
  where $p_{i}^{\prime }={\left(u_{i},v_{i},w_{i}\right)}^{T}$ denotes the projected point cloud coordinates.
  Step 5: The maximum and minimum values along the three principal axis directions are determined, respectively. The body center point of the standard segment in the local principal axis coordinate system is given by Equation (7):
  (7) $$c_{j}^{\prime }={\left(\frac{u_{\max}+u_{\min}}{2},\frac{v_{\max}+v_{\min}}{2},\frac{w_{\max}+w_{\min}}{2}\right)}^{T}$$
  where $c_{j}^{\prime }$ denotes the body center point coordinates of the j-th standard segment in the local principal axis coordinate system.
  Step 6: The body center point in the local coordinate system is transformed back to the original coordinate system to obtain the body center point coordinates of the standard segment, as expressed in Equation (8):
  (8) $$c_{j}=\bar{p}+Rc_{j}^{\prime }={\left(x_{c},y_{c},z_{c}\right)}^{T}$$
  where $c_{j}$ denotes the body center point coordinates of the j-th standard segment in the original coordinate system.
  Step 7: For the m standard segments of the square tower structure, m body center point coordinates are obtained, and the corresponding body center point set is given by Equation (9):
  (9) $$C=\left\{c_{1},c_{2},\cdots ,c_{m}\right\}$$
  where C denotes the set of body center points of the m standard segments of the square tower structure.

#### 2.1.3. Construction of the Dual-Cuboid Constraint Frame

Taking the body center point of the standard segment as the reference, the body center point of the standard segment point cloud is used as the alignment reference. This paper constructs an outer cuboid constraint frame and an inner cuboid constraint frame in the local coordinate system to perform spatial filtering of the standard segment point cloud. The outer cuboid constraint frame is used to enclose the overall spatial range of the standard segment, while the inner cuboid constraint frame is used to remove the internal point cloud of the structure. The point cloud that simultaneously satisfies the conditions of “being inside the outer cuboid constraint frame” and “not being inside the inner cuboid constraint frame” is defined as the shell point cloud of the standard segment.

First, the outer cuboid constraint frame is constructed. Taking the body center point $o(x_{o},y_{o},z_{o})$ of the standard segment as the reference, the outer cuboid constraint frame is established in the local coordinate system. The point cloud that satisfies the spatial constraint conditions of the outer cuboid constraint frame is defined as the inner point cloud set of the outer frame. The inner point cloud set of the outer cuboid constraint frame is given by Equation (10):

(10) $$\begin{array}{l} P_{outer}=\\ \left\{p_{i}^{outer}(x_{i}^{outer},y_{i}^{outer},z_{i}^{outer})\in P\left|\left|x_{i}^{outer}-x_{o}\right|\leq \frac{a+\varepsilon }{2},\left|y_{i}^{outer}-y_{o}\right|\leq \frac{b+\varepsilon }{2},\left|z_{i}^{outer}-z_{o}\right|\leq \frac{c+\varepsilon }{2}\right.\right\} \end{array}$$

where $P_{outer}$ is the point cloud set inside the outer cuboid constraint frame, $x_{i}^{outer}$ is the coordinate value of the i point in the outer cuboid constraint frame point cloud set along the x direction, $y_{i}^{outer}$ is the coordinate value of the i point along the y direction, and $z_{i}^{outer}$ is the coordinate value of the i point along the z direction. $x_{o}$, $y_{o}$,and $z_{o}$ are the coordinate values of the body center point o of the standard segment along the x, y, and z directions, respectively; a, b, and c are the length, width, and height of the standard segment, respectively; and ε is the dimensional error term.

Next, the inner cuboid constraint frame is constructed. Taking the same body center point $o(x_{o},y_{o},z_{o})$ of the standard segment as the reference, the inner cuboid constraint frame is established in the local coordinate system. The point cloud that satisfies the spatial constraint conditions of the inner frame is defined as the point cloud set inside the inner cuboid constraint frame, denoted as $P_{inner}$. The point cloud set inside the inner cuboid constraint frame is given by Equation (11):

(11) $$\begin{array}{l} P_{inner}=\\ \left\{p_{i}^{inner}(x_{i}^{inner},y_{i}^{inner},z_{i}^{inner})\in P\left|\left|x_{i}^{inner}-x_{o}\right|\leq \frac{a-\varepsilon }{2},\left|y_{i}^{inner}-y_{o}\right|\leq \frac{b-\varepsilon }{2},\left|z_{i}^{inner}-z_{o}\right|\leq \frac{c-\varepsilon }{2}\right.\right\} \end{array}$$

where $P_{inner}$ is the point cloud set inside the inner cuboid constraint frame, $x_{i}^{inner}$ is the coordinate value of the i point in the inner cuboid constraint frame point cloud set along the x direction, $y_{i}^{inner}$ is the coordinate value of the i point along the y direction, and $z_{i}^{inner}$ is the coordinate value of the i point along the z direction.

Then, the finally retained shell point cloud can be expressed as Equation (12).

(12) $$P_{shell}=P_{outer}-P_{inner}$$

where $P_{shell}$ is the shell point cloud set of the standard segment.

#### 2.1.4. Marching Cubes-Based Extraction of Three-Dimensional Contour Vertices from Constrained Shell Point Clouds

The Marching Cubes algorithm is a classical method for three-dimensional isosurface reconstruction. Its fundamental principle is to search for an isosurface voxel by voxel within a regular voxel grid. Based on the relationship between the scalar values at the voxel vertices and a prescribed isovalue, local triangular meshes are constructed within individual voxels and subsequently assembled to form a continuous three-dimensional isosurface model. Owing to its stable topology, high computational efficiency, and satisfactory preservation of geometric details, the Marching Cubes algorithm has been widely applied in medical image reconstruction, industrial three-dimensional measurement, reverse engineering, and point cloud surface reconstruction.

In this study, the standard-segment shell point clouds extracted using the dual-cuboid constraint frame were used as the input data. The discrete point clouds were first voxelized, after which the Marching Cubes algorithm was employed to reconstruct the external three-dimensional surface models of the standard segments. Three-dimensional contour lines were subsequently extracted from the reconstructed surfaces, providing continuous and stable spatial features with complete geometric representation for the subsequent calculation of the body center points of the standard segments and fitting of the central axis.

The Marching Cubes algorithm first partitions the three-dimensional space into a regular voxel grid, in which each voxel consists of eight vertices. A scalar field is constructed by computing, for each voxel vertex, the Euclidean distance to its nearest point in the constrained shell point cloud. Thus, the scalar value assigned to each voxel vertex encodes the proximity of that vertex to the structural surface. The scalar values corresponding to the eight vertices of a given voxel are defined in Equation (13):

(13) $$s_{1},s_{2},\cdots ,s_{8}$$

The isovalue was set adaptively based on the average point spacing of the input shell point cloud, following the approach commonly used in point-based isosurface extraction. Specifically, the isovalue was chosen as a small fraction of the mean nearest-neighbor distance of the shell point cloud, ensuring that the extracted contour faithfully represents the external surface geometry while avoiding topological artifacts. Given a prescribed isovalue τ, whether the isosurface intersects the current voxel can be determined by comparing the scalar value at each vertex with the isovalue. When the scalar values at the two endpoints of a voxel edge fall on opposite sides of the prescribed isovalue, the isosurface is considered to intersect that edge, and the position of the intersection point must be calculated. Let the coordinates of the two endpoints of the voxel edge be $q_{1}$ and $q_{2}$, with the corresponding scalar values denoted by $s_{1}$ and $s_{2}$, respectively. The coordinates P of the intersection point can then be obtained by linear interpolation, as expressed in Equation (14):

(14) $$q_{iso}=q_{1}+\frac{\tau -s_{1}}{s_{2}-s_{1}}\left(q_{2}-q_{1}\right)$$

where $q_{iso}$ denotes the coordinates of the intersection point between the isosurface and the current voxel edge; τ denotes the prescribed isovalue; $s_{1}$ and $s_{2}$ denote the scalar values corresponding to the two endpoints of the voxel edge; and $q_{1}$ and $q_{2}$ denote the coordinates of the two endpoints, respectively.

To ensure the smoothness of the reconstructed surface, linear interpolation was also employed to calculate the normal vector at each intersection point. Let $m_{1}$ and $m_{2}$ denote the normal vectors corresponding to the two endpoints of a voxel edge. The interpolated normal vector at the intersection point is expressed in Equation (15):

(15) $$m_{iso}=m_{1}+\frac{\tau -s_{1}}{s_{2}-s_{1}}\left(m_{2}-m_{1}\right)$$

where $m_{iso}$ denotes the normal vector at the intersection point; $m_{1}$ and $m_{2}$ denote the normal vectors corresponding to the two endpoints of the voxel edge.

For each voxel, each of its eight corner nodes can be classified into one of two states according to whether its scalar value is below or above the isovalue, corresponding to the two sides of the isosurface. Therefore, a voxel has $2^{8}=256$ possible topological configurations. After rotational and reflectional symmetries of the cube are taken into consideration, these configurations can be further reduced to 15 basic topological cases. The Marching Cubes algorithm uses a predefined lookup table to rapidly determine the triangle connection pattern corresponding to the current voxel and constructs the local triangular mesh according to a consistent topological rule. As all voxels are traversed, the local triangular facets are progressively assembled to form a complete and spatially continuous three-dimensional isosurface model.

**Figure 2:**
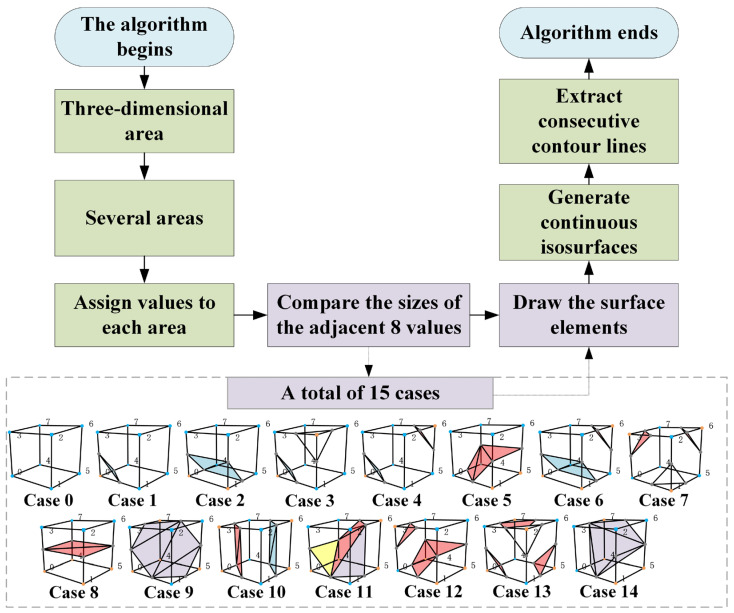
Contour line extraction process for transverse cuboid point clouds based on the Marching Cubes algorithm.

The Marching Cubes algorithm was employed to extract the contour lines of the transversely segmented cuboid point clouds of the tower body. To accommodate the transverse cuboid point cloud segmentation obtained under different strategy schemes, a contour line extraction method based on the Marching Cubes algorithm was developed for the transversely segmented cuboid point clouds of the tower body. The corresponding extraction workflow is illustrated in Figure 2.

Based on the vertices of the contour lines extracted from the cuboid shell point clouds, the updated contour-line center points were calculated using the principal axis projection method, as expressed in Equation (16):

(16) $$O=\left\{o_{1},o_{2},\cdots ,o_{m}\right\}$$

where O denotes the set of body center points corresponding to the m cuboid point cloud contour lines.

The dual-cuboid constraint frame and the Marching Cubes algorithm perform two distinct but sequential functions in the proposed workflow. First, the dual-cuboid constraint frame is applied to each standard segment point cloud to define a dimension-guided admissible spatial domain. Through outer-frame enclosure and inner-frame exclusion, the original standard segment point cloud is spatially filtered to obtain the shell point cloud $P_{shell}$. Therefore, the dual-cuboid constraint frame determines which points are allowed to enter the subsequent geometric feature extraction procedure.

The Marching Cubes algorithm does not participate in the spatial filtering of the original point cloud. Instead, it converts the irregularly distributed constrained shell point cloud into a scalar field defined on a regular voxel grid and extracts the corresponding isosurface. The specific information obtained using the Marching Cubes algorithm includes the interpolated isosurface vertex set and the corresponding triangle connectivity. In the subsequent verticality inspection procedure, the isosurface vertices are used to calculate the body center point of each standard segment, whereas the triangle connectivity is retained solely to represent the local topological continuity of the reconstructed surface. Therefore, the dual-cuboid constraint frame provides the geometric input for the Marching Cubes algorithm, while the Marching Cubes algorithm transforms the constrained shell point cloud into an explicit three-dimensional contour representation suitable for body center point calculation and central axis fitting.

The Marching Cubes algorithm was selected in this study because it is compatible with the three-dimensional characteristics of the constrained shell point cloud and directly provides the interpolated isosurface vertices required for body center point calculation. Unlike the Marching Square algorithm, which extracts independent two-dimensional contours from selected planar slices, the Marching Cubes algorithm processes the complete three-dimensional voxelized shell point cloud of each standard segment while preserving the spatial connectivity among neighboring voxel cells. Compared with Delaunay-based alpha-shape reconstruction [59], the Marching Cubes approach avoids the construction of a global Delaunay triangulation and enables the extracted geometric details to be controlled through the voxel spacing and isovalue. Compared with Poisson surface reconstruction [60], which formulates surface reconstruction as a global problem using oriented points, the present approach requires only a local distance scalar field and lookup-table-based interpolation to obtain the contour vertices required for subsequent body center point calculation. Therefore, it provides a direct and controllable interface between the shell point cloud extracted using the dual-cuboid constraint frame and the subsequent calculations of the body center points and central axis.

#### 2.1.5. Determination of the Spatial Line Equation and Direction Vector of the Central Axis of Square Tower Structures

For the m body center points of the corresponding cuboid point clouds in set O, a procedure was established to determine the direction vector of the spatial line representing the central axis of the square tower structure. In this study, the spatial line equation of the central axis was fitted using the least squares method, and the corresponding direction vector was subsequently calculated. The specific procedure is described as follows.

First, the point–direction form of the spatial line equation representing the central axis of the square tower structure is expressed as Equation (17):

(17) $$\frac{x-x_{0}}{p_{1}}=\frac{y-y_{0}}{p_{2}}=\frac{z-z_{0}}{p_{3}}$$

where $p_{1}$, $p_{2}$ and $p_{3}$ denote the components of the direction vector of the spatial line representing the central axis of the square tower structure along the three coordinate axes, respectively, and $\left(x_{0},y_{0},z_{0}\right)$ denotes the coordinates of an arbitrary known point on this spatial line. Equation (17) can be equivalently transformed into Equation (18):

(18) $$\left.\begin{array}{l} x=\frac{p_{1}}{p_{3}}\cdot \left(z-z_{0}\right)+x_{0}=k_{1}\cdot z+b_{1}\\ y=\frac{p_{2}}{p_{3}}\cdot \left(z-z_{0}\right)+y_{0}=k_{2}\cdot z+b_{2} \end{array}\right\}$$

where $k_{1}=\frac{p_{1}}{p_{3}}$,$b_{1}=x_{0}-\frac{p_{1}}{p_{3}}\cdot z_{0}$,$k_{2}=\frac{p_{2}}{p_{3}}$,$b_{2}=y_{0}-\frac{p_{2}}{p_{3}}\cdot z_{0}$.

Next, let $x_{j}^{\prime }=k_{1}\cdot z_{j}+b_{1}$ and $x_{j}^{\prime }=k_{2}\cdot z_{j}+b_{2}$ denote the fitted coordinate values $\left(x_{j}^{\prime },y_{j}^{\prime }\right)$ obtained from Equation (18). The sums of the squared residuals between the fitted coordinate values and the observed coordinate values $\left(x_{j},y_{j}\right)$, respectively, are expressed in Equation (19):

(19) $$\left.\begin{array}{l} Q_{1}=\sum_{j=1}^{m}(x_{j}-{x^{\prime }}_{j}{)}^{2}=\sum_{j=1}^{m}(x_{j}-k_{1}z_{j}-b_{1}{)}^{2}\\ Q_{2}=\sum_{j=1}^{m}(y_{j}-{y^{\prime }}_{j}{)}^{2}=\sum_{j=1}^{m}(y_{j}-k_{2}z_{j}-b_{2}{)}^{2} \end{array}\right\}$$

According to the fitting principle of least squares indirect adjustment, $Q_{1}$ and $Q_{2}$ should be minimized. To obtain their minimum values, Equation (19) is differentiated with respect to $k_{1}$, $b_{1}$, $k_{2}$, and $b_{2}$, respectively, and the resulting derivatives are set equal to zero, as expressed in Equation (20):

(20) $$\left.\begin{array}{l} b_{1}m+k_{1}\sum_{j=1}^{m}z_{j}=\sum_{j=1}^{m}x_{j},b_{1}\sum_{j=1}^{m}z_{j}+k_{1}\sum_{j=1}^{m}z_{j}^{2}=\sum_{j=1}^{m}x_{j}z_{j}\\ b_{2}m+k_{2}\sum_{j=1}^{m}z_{j}=\sum_{j=1}^{m}y_{j},b_{2}\sum_{j=1}^{m}z_{j}+k_{2}\sum_{j=1}^{m}z_{j}^{2}=\sum_{j=1}^{m}y_{j}z_{j} \end{array}\right\}$$

The parameters $k_{1}$, $b_{1}$, $k_{2}$, and $b_{2}$ can then be obtained from Equation (20), as expressed in Equation (21):

(21) $$\left.\begin{array}{l} k_{1}=\frac{m\sum_{j=1}^{m}x_{j}\cdot z_{j}-\sum_{j=1}^{m}x_{j}\cdot \sum_{j=1}^{m}z_{j}}{m\sum_{j=1}^{m}z_{j}^{2}-\sum_{j=1}^{m}z_{j}\cdot \sum_{j=1}^{m}z_{j}},b_{1}=\frac{\sum_{j=1}^{m}x_{j}-k_{1}\sum_{j=1}^{m}z_{j}}{m}\\ k_{2}=\frac{m\sum_{j=1}^{m}y_{j}\cdot z_{j}-\sum_{j=1}^{m}y_{j}\cdot \sum_{j=1}^{m}z_{j}}{m\sum_{j=1}^{m}z_{j}^{2}-\sum_{j=1}^{m}z_{j}\cdot \sum_{j=1}^{m}z_{j}},b_{2}=\frac{\sum_{j=1}^{m}y_{j}-k_{2}\sum_{j=1}^{m}z_{j}}{m} \end{array}\right\}$$

where $\left(x_{j},y_{j},z_{j}\right)$ denotes the coordinates of the body center point corresponding to the j-th cuboid point cloud contour line.

Finally, an arbitrary value is assigned to $\forall z_{0}$, and the corresponding $x_{0}$ and $y_{0}$ are calculated using $k_{1}$, $b_{1}$, $k_{2}$, and $b_{2}$ obtained from Equation (21). Similarly, an arbitrary value is assigned to $\forall p_{3}$, and the corresponding $p_{1}$ and $p_{2}$ are calculated using $k_{1}$ and $k_{2}$ in Equation (18). Accordingly, the fitted spatial line equation in point–direction form is established, and the direction vector of the spatial line representing the central axis of the square tower structure is determined, as expressed in Equation (22):

(22) $$\vec{M}=\left[p_{1},p_{2},p_{3}\right]$$

where M denotes the direction vector of the spatial line representing the central axis of the square tower structure.

According to Equation (22), the direction vector $\vec{M}$ is normalized to obtain the unit direction vector of the spatial line representing the central axis of the square tower structure, as expressed in Equation (23):

(23) $${\vec{M}}^{0}=\left[p_{1}^{0},p_{2}^{0},p_{3}^{0}\right]$$

where $M^{0}$ denotes the normalized direction vector of the spatial line representing the central axis of the square tower structure.$p_{1}^{0}=p_{1}/\sqrt{{\left(p_{1}\right)}^{2}+{\left(p_{2}\right)}^{2}+{\left(p_{3}\right)}^{2}}$, $p_{2}^{0}=p_{2}/\sqrt{{\left(p_{1}\right)}^{2}+{\left(p_{2}\right)}^{2}+{\left(p_{3}\right)}^{2}}$, $p_{3}^{0}=p_{3}/\sqrt{{\left(p_{1}\right)}^{2}+{\left(p_{2}\right)}^{2}+{\left(p_{3}\right)}^{2}}$.

#### 2.1.6. Calculation of Verticality-Related Attitude Parameters of the Central Axis of Square Tower Structures

1. Calculate the azimuth angle of inclination of the central axis of the square tower structure. In the terrestrial LiDAR station-centered spatial coordinate system, the unit vector of the x-axis is $\vec{X}=\left[1,0,0\right]$. Then, the azimuth angle of inclination α of the central axis of the square tower structure is obtained by vector operation between d and $\vec{X}$, as shown in Equation (24):
  (24) $$\alpha =\arccos\left(\frac{\vec{X}\cdot d}{\left|\vec{X}\right|\cdot \left|d\right|}\right)$$
2. Calculate the inclination angle of the central axis of the square tower structure. In the terrestrial LiDAR station-centered spatial coordinate system, the unit vector of the z-axis is $\vec{Z}=\left[0,0,1\right]$. Then, the inclination angle θ of the central axis of the square tower structure is obtained by vector operation between d and $\vec{Z}$, as shown in Equation (25):
  (25) $$\theta =\arccos\left(\frac{\vec{Z}\cdot d}{\left|\vec{Z}\right|\cdot \left|d\right|}\right)$$
3. Calculate the verticality of the square tower structure. According to Equation (25), the verticality of the square tower structure is given by Equation (26):
  (26) I=tanθ

#### 2.1.7. Accuracy Evaluation of Verticality-Related Attitude Parameters

To quantitatively compare the degree of difference among verticality inspection results obtained by different point cloud processing methods, the relative error metric is adopted in this paper to conduct comparative analysis of the calculation results of each method.

The calculation formula of the relative error metric is given by Equation (27):

(27) $$\varphi =\frac{\left|I_{T}-I_{R}\right|}{\left|I_{R}\right|}\times 100\%$$

where φ is the relative error; $I_{T}$ is the verticality inspection result obtained by the method to be compared; and $I_{R}$ is the result obtained by the experimental reference method.

### 2.2. Materials

The experimental object of this paper is a square tower structure, characterized by a regular cross-sectional shape, clear edges, and a distribution of repeated stacking of standard segments along the height direction. A tower crane of a certain brand at a construction site in Beijing is selected as the research object. Its tower body consists of multiple standard segments, and the three-dimensional structural characteristics of each standard segment together constitute the overall square geometric outer contour of the tower body. The standard segment is the basic unit of the square tower structure and the main carrier for the formation and transmission of the overall verticality characteristics. Although a tower crane with a quadrilateral cross-section is used as an example for method validation in this paper, the proposed algorithm is, in principle, also applicable to square tower structures with regular cross-sections and clear three-dimensional contours, demonstrating good applicability and extensibility.

In addition, during hoisting operations, tower cranes are often subjected to complex working conditions such as hoisting loads, wind loads, and slewing moments, which can easily cause geometric attitude changes such as structural inclination and joint offsets. The verticality state of the tower crane is directly related to construction safety and equipment stability. Therefore, selecting the tower crane as the experimental object and data material for this paper not only provides clear geometric structural characteristics but also fully verifies the effectiveness and engineering applicability of the proposed method in verticality inspection.

## 3. Experiment

### 3.1. Experimental Design

To verify and analyze the feasibility and effectiveness of the proposed dual-cuboid constraint method for inspecting the verticality of square tower structures using the Marching Cubes algorithm, the functional modules of the proposed method were implemented in MATLAB R2024a. A tower crane located at a construction site in Beijing was selected as the experimental object. The tower crane had an overall height of approximately 63 m and comprised three components: the upper structure, with a height of approximately 23 m; the tower body, with a height of approximately 35 m; and the base, with a height of approximately 5 m.

A RIEGL VZ-600i terrestrial LiDAR scanner (manufactured by RIEGL LMS GmbH, Horn, Austria) was employed for field data acquisition. The main specifications of the scanner included a measurement range of 0.5–1000 m, a three-dimensional position accuracy of 3 mm at 50 m and 5 mm at 100 m, a field of view of 360° × 105°, a vertical angular resolution of less than 0.0007°, a horizontal angular resolution of less than 0.0005°, a three-dimensional coordinate measurement accuracy of (5.1, 5.1, 1.2) mm at 100 m, and a data acquisition rate of 2.2 million points/s. Regarding scanner performance, in Panorama 6 mm mode, each scan requires less than 30 s and generates approximately 3 million points, with a spatial resolution of 6 mm at a distance of 10 m. The system supports up to 60 scanning stations per hour, including scanning and image acquisition, together with on-board real-time automatic registration. This mode is intended for low-resolution overview scanning, with a nominal single-station scanning time of 30 s. The laser pulse repetition rate is up to 2.2 MHz. Eight scanning stations were established at suitable locations approximately 100–150 m from the tower crane, each providing an unobstructed view of the structure. Full-surround scanning measurements were conducted to acquire the raw point cloud data of the tower crane, including the raw point cloud data of the tower body. The corresponding measurement accuracy was approximately 7.3–10.97 mm.

**Figure 3:**
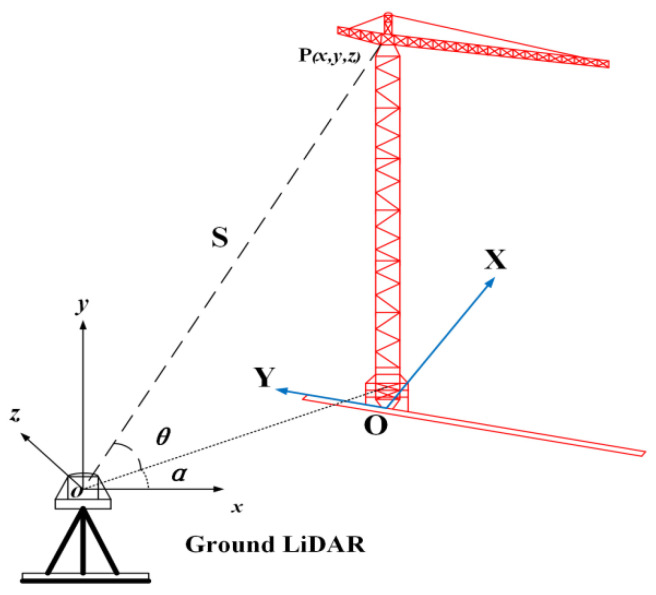
Diagram of experimental test.

The terrestrial LiDAR scanner was freely stationed at suitable locations within the construction site. The *o-xyz* coordinate system was defined as the station-centered spatial coordinate system of the terrestrial LiDAR scanner, whereas the O-XY coordinate system was defined as the planar coordinate system of the tower body. The software supplied with the RIEGL VZ-600i (RiSCAN PRO) was used for inter-station point cloud registration, coordinate transformation, and preprocessing, including redundancy removal and denoising. Consequently, effective high-precision point cloud data of the tower body were obtained in the unified terrestrial LiDAR station-centered spatial coordinate system. A schematic diagram of the experimental test is presented in Figure 3.

All algorithmic modules of the proposed method, including point cloud segmentation, PCA-based body center point calculation, dual-cuboid constraint filtering, Marching Cubes-based contour extraction, and least squares central axis fitting, were implemented in MATLAB R2024a. The experiments were performed on a computer equipped with an Intel Core i5-11260H processor (manufactured by Intel Corporation, Santa Clara, CA, USA) operating at 2.60 GHz and 16 GB of memory. For the tower-crane dataset used in this study, which contained approximately 1.15 million valid tower-body points distributed across six standard segments, the total processing time was approximately 45–60 s. Point cloud segmentation and PCA-based body center point calculation required approximately 3–5 s, dual-cuboid constraint filtering required approximately 15–25 s, Marching Cubes-based contour extraction required approximately 15–20 s, and least squares central axis fitting required less than 5 s. Constraint filtering and contour extraction accounted for most of the total processing time because these procedures involved pointwise spatial comparisons and voxel-based interpolation. These results demonstrate the computational feasibility of the proposed method for routine engineering inspection datasets of a comparable scale.

### 3.2. Preprocessing of LiDAR Point Cloud Data

**Figure 4:**
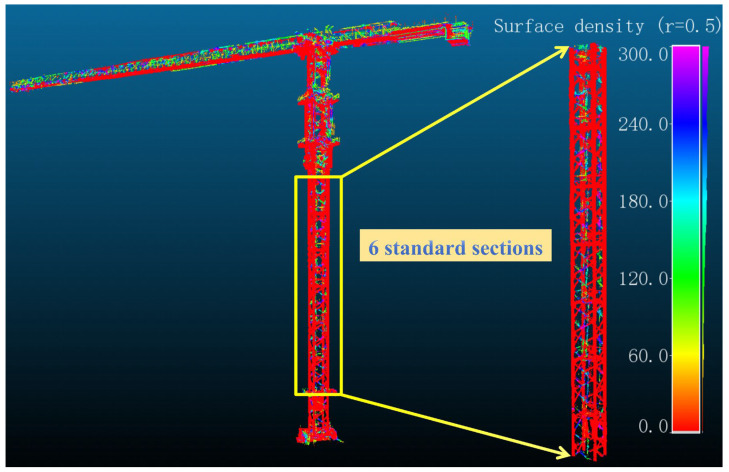
Effective point cloud data of tower crane body.

The accompanying software for the RIEGL VZ-600i was used for point-cloud registration and denoising, and the processed data were exported in LAS format. During registration, three stable natural feature points were selected as corresponding points to align the multi-station data within a unified coordinate system. The valid point-cloud data of the tower crane body are shown in Figure 4.

As shown in Figure 4, the proposed method focuses on the tower crane body. Before preprocessing, the point cloud data of the tower crane also contained interfering data from the jib, top, and base. Subsequently, based on the high-precision point cloud data in LAS format, CloudCompare v 2.14 alpha was used to perform point cloud segmentation to extract the valid point cloud data of the tower crane body. A statistical outlier removal filter was subsequently applied to eliminate isolated noise points, with the number of neighboring points set to 50 and the standard deviation multiplier set to 1.5. The merged point cloud before preprocessing contained approximately 8.5 million points. Approximately 1.20 million points remained after target-region segmentation, and approximately 1.15 million valid tower-body points were ultimately retained after denoising, corresponding to a point cloud retention ratio of approximately 13.5%. The preprocessed point cloud had an average point density of 3200 points/m^2^ and a mean nearest-neighbor spacing of approximately 17.7 mm. Ultimately, the valid point cloud data of the independent section of the tower crane body, which consists of six standard segments, were obtained.

It should be noted that manual segmentation of the complete-scene point cloud was performed using CloudCompare solely to extract the tower-body region from the overall point cloud of the tower crane as the research object and to remove interfering data associated with the jib, tower top, and tower base. This procedure involved only the extraction of the target region and did not include any modification of point cloud coordinates, filtering, resampling, or geometric correction. Therefore, the original spatial geometric information of the tower-body point cloud was fully preserved.

After the tower body had been extracted, it was vertically partitioned into six standard segments, each with a height of 5.70 m, according to the actual structural dimensions of the standard segments of the tower crane. These standard segments were subsequently treated as individual objects in the experiments. All procedures proposed in this study, including the construction of the dual-cuboid constraint frame, extraction of the shell point cloud, calculation of the body center points, fitting of the central axis, and calculation of verticality, were performed automatically and directly using the original point cloud data of each standard segment without manual intervention.

Accordingly, manual segmentation was used solely to define the spatial extent of the research object and did not alter the spatial positions or geometric relationships of the retained point cloud. Therefore, it had no influence on point cloud quality, body center point extraction, central axis fitting, or the final verticality calculation results. Furthermore, owing to the clearly defined boundaries of the tower body and the fixed dimensions of the standard segments, the target-region extraction procedure exhibited good repeatability. Consistent tower-body point cloud data could be obtained by different operators using the same structural boundaries.

### 3.3. Design of Horizontal Slicing Schemes for Point-Cloud Segmentation

To compare and analyze the effects of transverse cuboid point cloud segmentation under different schemes on the verticality inspection results of the tower body, seven point cloud processing schemes with different dual-cuboid constraint-frame dimensional parameters were designed according to the number of standard segments in the tower body of the tower crane. Using the CloudCompare software platform, the uppermost standard segment of the tower body was designated as the first standard segment. Among the seven schemes, Scheme A, Scheme B, Scheme C, Scheme D, and Scheme E were designed based on the method proposed in this study. Specifically, dual-cuboid constraint frames with dimensional errors of 20 mm, 40 mm, 60 mm, and 80 mm were adopted in Scheme A, Scheme B, Scheme C, and Scheme D, respectively. In Scheme E, no dual-cuboid constraint frame was applied, whereas all other processing procedures were identical to those of the proposed method; therefore, Scheme E was used as a comparative experimental scheme. Scheme F was established according to the Marching Square algorithm method reported in Reference [30]. Because the feasibility and effectiveness of the method had previously been validated through comparisons with conventional inspection methods, Scheme F was adopted as a literature-based reference experimental scheme. Similarly, Scheme G was established according to the RANSAC algorithm method reported in Reference [61], whose feasibility and effectiveness had also been demonstrated in the corresponding study. Scheme F and Scheme G were introduced to strengthen the rationale underlying the experimental design adopted in this study.

To verify the rationality of the dimensional parameters of the different dual-cuboid constraint frames, a sensitivity analysis of the constraint-frame dimensions was further conducted. The dimensional error between the actual dimensions of the inner and outer cuboid constraint frames was treated as the variable, and four constraint conditions with dimensional errors of 20 mm, 40 mm, 60 mm, and 80 mm were established. The shell point cloud extraction performance and the final verticality results obtained under the four conditions were comparatively analyzed. The dimensional error parameters adopted in this study were determined through comprehensive consideration of the following three factors. First, regarding instrument measurement accuracy, the overall measurement accuracy of the RIEGL VZ-600i at scanning distances of 100–150 m is approximately 7.3–10.97 mm. Therefore, the lower bound of the dimensional error parameter should be substantially greater than the instrument measurement error to ensure that the constraint results reflect the geometric characteristics of the structure rather than measurement noise. Second, regarding the actual dimensions of the connecting components between standard segments, adjacent tower-crane standard segments are connected by components such as connection plates and pins, which protrude to a certain extent from the surfaces of the main chords. The dimensional error parameter must therefore be greater than the protrusion dimensions of these connecting components to preserve the complete shell geometry. Third, regarding the range of engineering geometric uncertainties, factors such as residual point cloud registration errors and accumulated errors arising from multi-station registration were comprehensively considered. A range of 20–80 mm was considered sufficient to accommodate the various geometric uncertainties that may occur in practical engineering applications. Accordingly, four dimensional error parameters of 20 mm, 40 mm, 60 mm, and 80 mm were selected to systematically investigate the influence and sensitivity of the constraint-frame dimensional parameters on shell point cloud extraction and the resulting verticality inspection results.

The experimental results demonstrated that, under all four dimensional-error conditions, the dual-cuboid constraint frames enabled complete extraction of the effective point clouds on the external surfaces of the standard segments while effectively removing redundant internal points. The resulting shell point clouds satisfactorily preserved the geometric characteristics of the standard segments. As the dimensional error increased, the number of retained shell points gradually increased, whereas only minor variations were observed in the final calculated verticality values. These results indicate that, within the dimensional-error range of 20–80 mm, variations in the constraint-frame dimensions exerted only a limited influence on the verticality inspection results, thereby demonstrating the satisfactory parameter robustness of the proposed dual-cuboid constraint method.

Furthermore, the measurement accuracy of the LiDAR scanning system employed in this study was 3–5 mm, whereas the dimensional errors of 20–80 mm were substantially greater than the instrumental measurement errors. Therefore, when the point cloud measurement errors and the dimensional effects of local components, such as the connection plates of the standard segments, were taken into consideration, the constraint frames corresponding to the different dimensional errors were capable of completely retaining geometrically meaningful external-surface points while preventing a large number of redundant internal points from participating in the subsequent calculations. Consequently, the stability of body center point extraction and central axis fitting was ensured.

Based on the number of standard segments in the tower body of the tower crane, the uppermost standard segment was designated as the first standard segment using the CloudCompare software platform, and six point cloud processing schemes with different dual-cuboid constraint-frame dimensional parameters were designed:

1. Scheme A: Using the proposed method, the 1st, 2nd, 3rd, 4th, 5th, and 6th standard segments are taken as the horizontal slicing cubic point clouds. Under the dual-cuboid constraint frame with a dimensional error of 20 mm, the coordinates of the center points of the corresponding shell point cloud contour lines of the standard segments are extracted to fit the spatial line equation of the tower body’s central axis, and its direction vector is calculated.
2. Scheme B: Using the proposed method, the 1st, 2nd, 3rd, 4th, 5th, and 6th standard segments are taken as the horizontal slicing cubic point clouds. Under the dual-cuboid constraint frame with a dimensional error of 40 mm, the coordinates of the center points of the corresponding shell point cloud contour lines of the standard segments are extracted to fit the spatial line equation of the tower body’s central axis, and its direction vector is calculated.
3. Scheme C: Using the proposed method, the 1st, 2nd, 3rd, 4th, 5th, and 6th standard segments are taken as the horizontal slicing cubic point clouds. Under the dual-cuboid constraint frame with a dimensional error of 60 mm, the coordinates of the center points of the corresponding shell point cloud contour lines of the standard segments are extracted to fit the spatial line equation of the tower body’s central axis, and its direction vector is calculated.
4. Scheme D: Using the proposed method, the 1st, 2nd, 3rd, 4th, 5th, and 6th standard segments are taken as the horizontal slicing cubic point clouds. Under the dual-cuboid constraint frame with a dimensional error of 80 mm, the coordinates of the center points of the corresponding shell point cloud contour lines of the standard segments are extracted to fit the spatial line equation of the tower body’s central axis, and its direction vector is calculated.
5. Scheme E: Using the proposed method, the 1st, 2nd, 3rd, 4th, 5th, and 6th standard segments are taken as the horizontal slicing cubic point clouds. Without applying a dual-cuboid constraint frame, the coordinates of the center points of the corresponding shell point cloud contour lines of the standard segments are extracted to fit the spatial line equation of the tower body’s central axis, and its direction vector is calculated.
6. Scheme F: Using the Marching Square algorithm [30] method, horizontal slicing is performed at the joints between the 1st, 2nd, 3rd, 4th, 5th, and 6th standard segments to obtain the cross-sectional polygonal slice point clouds. The geometric center coordinates of contour lines of the sliced point cloud are extracted to fit the spatial line equation of the tower body’s central axis, and its direction vector is calculated.
7. Scheme G: Using the Ransac algorithm [61] method, horizontal slicing is performed at the joints between the 1st, 2nd, 3rd, 4th, 5th, and 6th standard segments to obtain the cross-sectional polygonal slice point clouds. The geometric center coordinates of contour lines of the sliced point cloud are extracted to fit the spatial line equation of the tower body’s central axis, and its direction vector is calculated.

## 4. Results

### 4.1. The Result of the Point Cloud Segmentation Scheme

To improve the accuracy and noise resistance of point cloud processing for the tower crane standard segments, spatial filtering of the standard segment point clouds was performed during the preprocessing stage based on the construction method of the dual-cuboid constraint frame. Two cuboid constraint frames, an inner cuboid constraint frame and an outer cuboid constraint frame, were constructed with the center of each standard segment as the reference. Because the cuboid dimensions of each standard segment in the experimental object were 2.50 m × 2.50 m × 5.70 m, the dimensional error term ε between the actual dimensions of the inner and outer cuboid constraint frames was treated as the control variable. Four constraint conditions with dimensional errors of 20 mm, 40 mm, 60 mm, and 80 mm were established. Accordingly, the dimensions of the outer cuboid constraint frames were 2.51 m × 2.51 m × 5.71 m, 2.52 m × 2.52 m × 5.72 m, 2.53 m × 2.53 m × 5.73 m, and 2.54 m × 2.54 m × 5.74 m, respectively. The corresponding dimensions of the inner cuboid constraint frames were 2.49 m × 2.49 m × 5.69 m, 2.48 m × 2.48 m × 5.68 m, 2.47 m × 2.47 m × 5.67 m, and 2.46 m × 2.46 m × 5.66 m, respectively.

**Figure 5:**
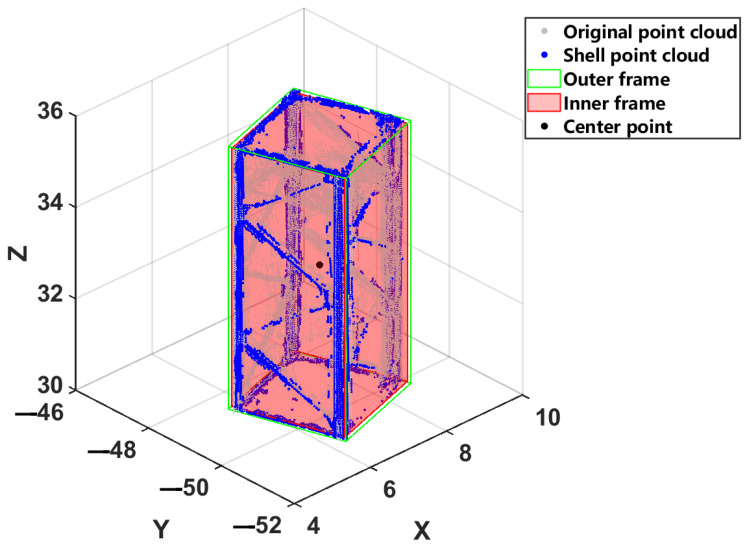
Schematic diagram of the dual-cuboid constraint frame.

Dual-cuboid constraint frames were constructed based on the different dimensional error terms to perform spatial constraint-based filtering of the standard segment point clouds and extract the corresponding shell point clouds. Under one of the dimensional-error conditions, the construction result of the dual-cuboid constraint frame for a representative standard segment is illustrated in Figure 5.

As shown in Figure 5, the green transparent cube represents the outer cuboid constraint frame, the red cube represents the inner cuboid constraint frame, and the blue point cloud is the finally retained shell point cloud. The cube contour lines are constructed based on the blue point cloud.

**Figure 6:**
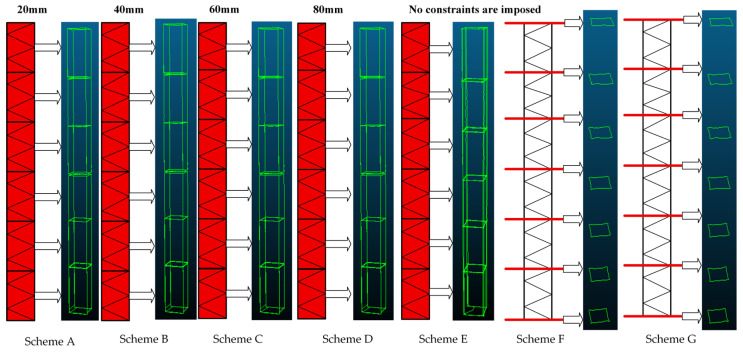
Seven experimental schemes and their corresponding segmentation results. The red regions indicate the point clouds to be extracted.

Scheme A, Scheme B, Scheme C, and Scheme D were designed as schemes incorporating additional constraints, whereas no dual-cuboid constraint frame was introduced in either Scheme E or Scheme F. Scheme E served as the comparative experiment, whereas Scheme F and Scheme G served as the reference experiment. Contour lines were extracted under all seven schemes, and the corresponding central axis fitting results are presented in Figure 6.

### 4.2. Verticality Results

**Figure 7:**
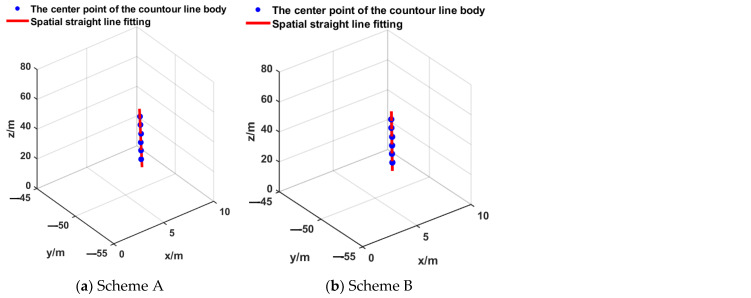

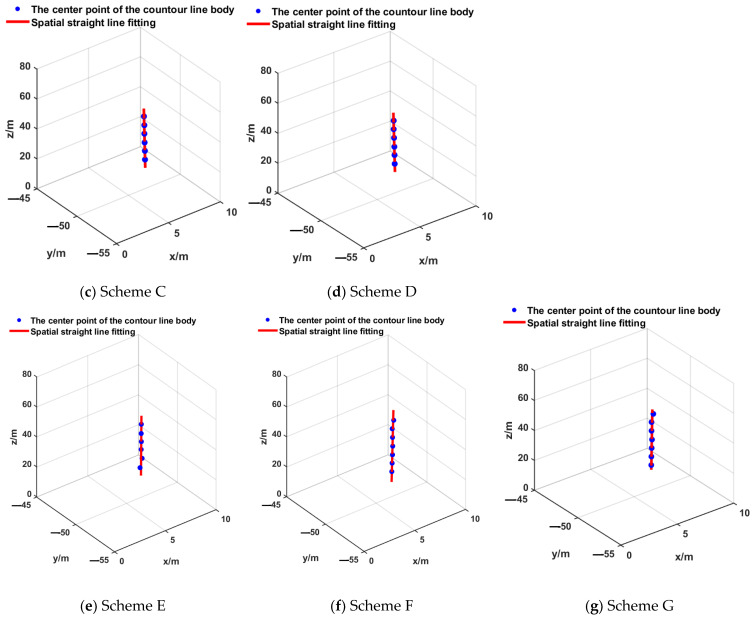
Spatial line of the tower central axis obtained from the seven schemes. (**a**) Results corresponding to Scheme A. (**b**) Results corresponding to Scheme B. (**c**) Results corresponding to Scheme C. (**d**) Results corresponding to Scheme D. (**e**) Results corresponding to Scheme E. (**f**) Results corresponding to Scheme F. (**g**) Results corresponding to Scheme G.

The central axis fitting results obtained using the proposed method for the first four schemes, along with the directly calculated central axis results for the latter three schemes, are shown in Figure 7.

Based on the spatial lines representing the central axis of the tower body obtained for the seven schemes shown in Figure 7, the corresponding direction vectors were determined. In the station-centered spatial coordinate system, vector operations were then performed between each direction vector and the x-axis unit vector $\left[1,0,0\right]$ and the z-axis unit vector $\left[0,0,1\right]$, respectively, to calculate the azimuth angle of inclination, inclination angle, and verticality of the tower body.

According to the performance specifications of the scanner, an uncertainty estimate of ±0.015‰ was obtained using the verticality error propagation formula. Since this uncertainty reflects the theoretical angular-resolution error of the scanner rather than the total uncertainty across different comparison schemes, it is not included in the table of tower-body verticality inspection results. The tower-body verticality inspection results for the seven schemes are presented in Table 1.

As shown in Table 1, six transversely segmented cuboid point clouds and six corresponding body center points were obtained for Scheme A. Using the proposed method, the tower-body verticality was calculated as 3.48‰, with an azimuth angle of inclination of 90°03′20″ and an inclination angle of 0°11′57″. For Scheme B, six transversely segmented cuboid point clouds and six corresponding body center points were obtained. The tower-body verticality calculated using the proposed method was 3.63‰, with an azimuth angle of inclination of 89°56′48″ and an inclination angle of 0°13′32″. For Scheme C, six transversely segmented cuboid point clouds and six corresponding body center points were obtained. The tower-body verticality calculated using the proposed method was 3.60‰, with an azimuth angle of inclination of 89°57′24″ and an inclination angle of 0°12′22″. For Scheme D, six transversely segmented cuboid point clouds and six corresponding body center points were obtained. The tower-body verticality calculated using the proposed method was 3.82‰, with an azimuth angle of inclination of 89°57′49″ and an inclination angle of 0°13′07″. For Scheme E, six transversely segmented cuboid point clouds and six corresponding body center points were obtained. The tower-body verticality calculated using the proposed method was 3.03‰, with an azimuth angle of inclination of 90°09′30″ and an inclination angle of 0°10′25″. For Scheme F, seven horizontal point cloud slices and seven corresponding cross-sectional center points were obtained. Using the Marching Square algorithm method reported in Reference [30], the tower-body verticality was calculated as 3.14‰, with an azimuth angle of inclination of 90°10′17″ and an inclination angle of 0°10′48″. For Scheme G, seven horizontal point cloud slices and seven corresponding cross-sectional center points were obtained. Using the RANSAC algorithm method reported in Reference [61], the tower-body verticality was calculated as 2.82‰, with an azimuth angle of inclination of 90°09′10″ and an inclination angle of 0°09′50″.

### 4.3. Verticality Accuracy Analysis Results

The seven strategies designed in this paper all achieved satisfactory verticality inspection results for the tower crane body. To further evaluate and compare the accuracy of the verticality inspection results obtained by different schemes, the verticality results obtained by Scheme E, Scheme F and Scheme G were taken as reference values, and the relative error metric was adopted to conduct the accuracy analysis of the verticality of the tower body for the different schemes. The corresponding analysis results are shown in Table 2.

It should be noted that Schemes F and G use seven cross-sectional points (one at each joint between adjacent standard segments), whereas Schemes A–E use six standard-segment body center points (one per standard segment). While both sets of points cover the same overall height range of the tower body (35 m) and are used to fit the same central axis using the least squares method, the difference in the number of points may have a minor influence on the stability of the axis fitting. This influence should be considered when interpreting the relative errors reported in Table 2.

As shown in Table 2, the following observations can be made. (1) Four dual-cuboid constraint frame schemes with different dimensional errors were designed using the proposed method incorporating an additional dual-cuboid constraint frame, and cross-sectional slices of the tower-crane point cloud were extracted. The tower-body verticality values obtained using Scheme A, Scheme B, Scheme C, and Scheme D were 3.48‰, 3.63‰, 3.60‰, and 3.82‰, respectively, with a mean value of 3.63‰. These results were in good agreement with the verticality values of 3.03‰ and 3.14‰ obtained using Scheme E and Scheme F, respectively, and showed reasonable proximity to the result of 2.82‰ obtained using Scheme G. All the results satisfied the requirement specified in GB/T 5031-2019 (Tower Cranes), which stipulates that the verticality shall not exceed 4‰. This finding indicates that the verticality of the tested tower crane complied with the current construction safety inspection standard and that the tower crane exhibited satisfactory structural stability and operational safety. (2) When Scheme E was adopted as the reference, the relative errors corresponding to Scheme A, Scheme B, Scheme C, and Scheme D were 14.85%, 19.80%, 18.81%, and 26.07%, respectively. When Scheme F was adopted as the reference, the relative errors corresponding to Scheme A, Scheme B, Scheme C, and Scheme D were 10.82%, 15.61%, 14.65%, and 21.66%, respectively. When Scheme G was adopted as the reference, the relative errors corresponding to Scheme A, Scheme B, Scheme C, and Scheme D were 23.40%, 28.72%, 27.65%, and 35.46%, respectively. These results indicate that the introduction of the additional dual-cuboid constraint frame did not cause the calculated results to deviate substantially from the overall inclination state of the tower body reflected by the comparative and reference schemes. Instead, the stability of point cloud feature extraction and central axis fitting was improved while consistency among the results was maintained. In other words, Scheme A, Scheme B, Scheme C, and Scheme D showed good agreement with Scheme E, Scheme F, and Scheme G, demonstrating that the proposed method can consistently characterize the overall inclination of the tower body.

## 5. Discussion

The tower-body verticality values obtained for Scheme A, Scheme B, Scheme C, and Scheme D using the proposed method were 3.48‰, 3.63‰, 3.60‰, and 3.82‰, respectively. A comparison of the schemes incorporating dual-cuboid constraint frames with different dimensional errors showed that, when the dimensional errors were set to 20 mm, 40 mm, 60 mm, and 80 mm, the corresponding tower-body verticality values were 3.48‰, 3.63‰, 3.60‰, and 3.82‰, respectively, with a mean value of 3.63‰ and a range of only 0.34‰. Overall, the verticality results obtained under the four dimensional parameter settings were relatively close. In particular, the verticality values obtained for Scheme B and Scheme C were 3.63‰ and 3.60‰, respectively, with a difference of only 0.03‰. This finding further indicates that, within a reasonable dimensional-error range, variations in the dimensional parameters of the dual-cuboid constraint frame exerted only a limited influence on the calculated tower-body verticality, demonstrating the satisfactory parameter robustness of the proposed method.

**Figure 8:**
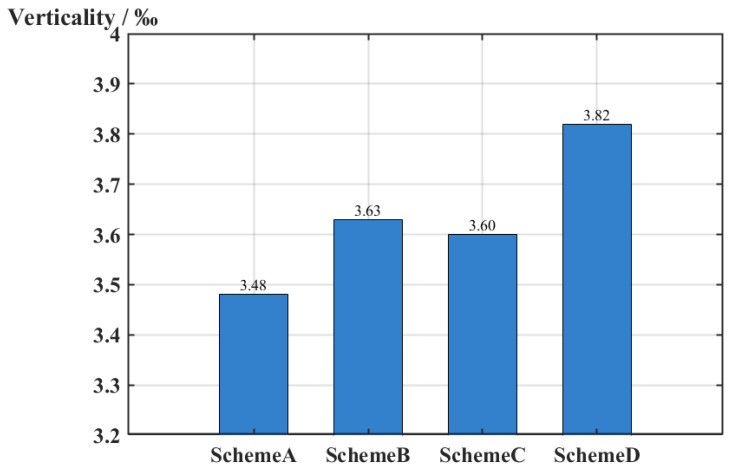
Variations in tower-body verticality under different dimensional-error conditions of the dual-cuboid constraint frame.

**Figure 9:**
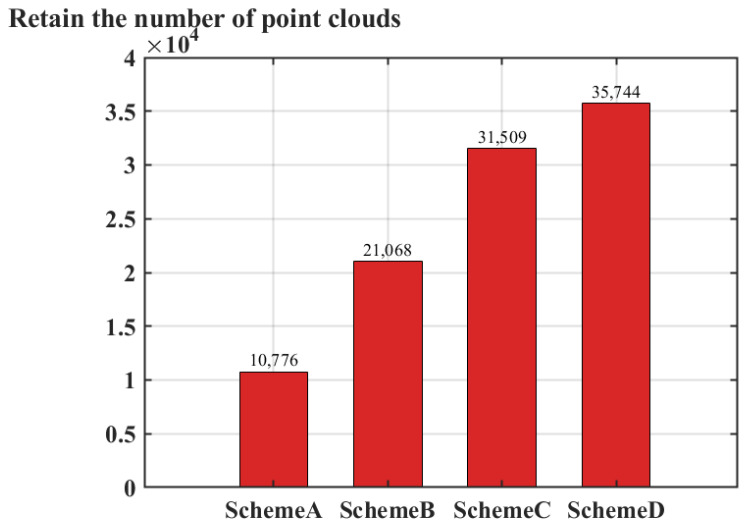
Variations in the number of retained shell point cloud points under different dimensional-error conditions of the dual-cuboid constraint frame.

The variations in tower-body verticality and the corresponding numbers of retained shell point cloud points obtained for Scheme A, Scheme B, Scheme C, and Scheme D under different dimensional-error conditions of the dual-cuboid constraint frame are presented in Figure 8 and Figure 9, respectively. By jointly examining these two figures, the effects of variations in the dimensional parameters of the dual-cuboid constraint frame on the verticality inspection results and the number of retained point cloud points can be further evaluated.

Further examination of the shell point cloud statistics revealed a continuous increase in the number of retained shell point cloud points as the dimensional error of the dual-cuboid constraint frame increased from 20 mm to 80 mm. When the dimensional error increased from 20 mm to 40 mm and 60 mm, the number of retained points increased from 10,776 to 21,068 and 31,509, respectively. These increases were substantial and broadly comparable. However, when the dimensional error was further increased from 60 mm to 80 mm, the number of retained points increased only to 35,744, indicating a marked reduction in the growth rate. This trend indicates that, as the constraint-frame dimensions increased, most of the newly retained point cloud points were introduced during the initial stages of dimensional expansion. Once the dimensional error reached 60 mm, most of the valid geometric points on the external shell of the standard segments had already been completely retained. Further enlargement of the constraint frame primarily introduced points from edge regions and a certain number of redundant points, which made only a limited contribution to the representation of the overall geometric characteristics of the standard segments. This also accounted for the relatively large differences between the tower-body verticality inspection result obtained for Scheme D and those obtained for Scheme A, Scheme B, and Scheme C. Therefore, for the experimental object investigated in this study, a dimensional error of 60 mm provided a satisfactory balance between the retention of valid geometric features and the control of redundant point cloud points and can thus be regarded as a preferable dimensional parameter for the dual-cuboid constraint frame.

Further comparison between the schemes incorporating dual-cuboid constraint frames and the two unconstrained comparison schemes showed that the tower-body verticality values obtained for Scheme A, Scheme B, Scheme C, and Scheme D were in good agreement with those obtained for Scheme E and Scheme F, and showed reasonable proximity to the result obtained for Scheme G, with no substantial deviation observed among the schemes. This finding indicates that the introduction of the dual-cuboid constraint frame did not alter the accurate representation of the overall inclination characteristics of the tower body. Instead, stable extraction of the shell point clouds of the standard segments was achieved while consistency among the overall verticality results was maintained. Compared with the unconstrained schemes, the schemes incorporating additional constraints enabled the effective removal of redundant internal points and local noise points while ensuring that the point cloud data used for body center point calculation were more concentrated within the actual external shell regions of the standard segments. Consequently, more stable geometric feature information was provided for subsequent central axis fitting.

From the perspective of the mechanisms underlying body center point calculation and central axis fitting, the stability of the tower-body verticality results primarily depended on the capability of the shell point clouds to represent the actual external geometry of the standard segments. The dual-cuboid constraint frame proposed in this study employed a spatial filtering strategy based on outer-frame enclosure and inner-frame exclusion. This strategy enabled complete retention of the shell point clouds of the standard segments while effectively suppressing the influence of redundant internal points, discrete noise points, and local outliers on body center point calculation. The experimental results demonstrated that, within the dimensional-error range of 20–80 mm, good consistency was maintained among the verticality results obtained using dual-cuboid constraint frames with different dimensional errors. These results indicate that the proposed dual-cuboid constraint method not only exhibits satisfactory parameter robustness but also improves the stability of body center point extraction and central axis fitting, thereby providing reliable geometric constraints for the verticality inspection of square tower structures.

In addition to the quantitative results and parameter sensitivity analysis presented above, several further aspects should be discussed to clarify the characteristics and limitations of the proposed method and its differences from existing approaches. First, in addition to instrument measurement errors and residual registration errors, the uniformity of point cloud density among different scanning stations may affect the verticality results. When the PCA-based OBB method is used to calculate body center points, it is assumed that the retained shell point cloud is uniformly distributed around the true geometric center. In practical scanning, however, point cloud densities may differ among the four faces of the tower body because of variations in scanning distance, incidence angle, and occlusion conditions. Such density differences may introduce systematic bias into the estimation of body center point positions. Although the dual-cuboid constraint reduces this effect to some extent by removing internal points and outliers, density-induced bias cannot be completely eliminated. This represents an inherent limitation of point-cloud-based geometric center estimation methods.

Second, although good agreement was obtained among the verticality results derived from the constrained schemes, unconstrained schemes, and reference methods, the absolute accuracy of the results could not be independently validated using conventional surveying instruments because the tower crane had already been dismantled after completion of the construction project. Therefore, the evaluation in this study was based on relative comparisons among different point cloud processing strategies rather than direct comparison with ground-truth measurements. This limitation of the current experimental design should be considered when interpreting the reported accuracy.

Third, compared with conventional TLS-based verticality inspection methods that rely on horizontal cross-sectional circle or ellipse fitting or cylindrical model fitting, a notable advantage of the proposed method is that no specific analytical shape needs to be assumed for each cross-section. Instead, the shell point clouds derived using the dual-cuboid constraint are directly processed using the PCA-based OBB method to calculate the body center points, thereby providing greater adaptability to the actual geometric morphology of the structure. However, this flexibility also results in greater sensitivity to the completeness of the shell point cloud. When one face of the tower body is incompletely scanned because of occlusion, the extracted body center point may shift toward the side with a higher point density. In contrast, cross-sectional fitting methods that impose a closed geometric shape may exhibit greater robustness to such localized data loss. This difference should be carefully considered when selecting an appropriate method for practical inspection applications. Overall, the proposed dual-cuboid constraint method demonstrated satisfactory consistency across the obtained results and robustness to parameter variations within its applicable scope. Nevertheless, its performance is affected by point cloud density uniformity, scanning completeness, and the availability of independent reference data. These considerations highlight the need for cautious interpretation of the experimental results and indicate specific directions for further methodological improvement and experimental validation.

## 6. Conclusions and Outlook

This paper proposes a Dual-Cuboid Constraint Method with Marching Cubes-Based Contour Extraction for Verticality Inspection of Square Tower Structures. The main conclusions are as follows:

1. The proposed LiDAR point cloud-based dual-cuboid constraint method enables stable modeling of the spatial morphology of the central axis of square tower structures and extraction of the associated tilt attitude parameters. The complete verticality inspection procedure, including high-precision point cloud acquisition, construction of the dual-cuboid constraint frame, calculation of body center points, and central axis fitting, is implemented, thereby providing a new technical approach for extracting the geometric attitude parameters of square tower structures. The dual-cuboid constraint frame is employed to spatially filter the point clouds of the standard segments, effectively retaining the structural shell point clouds while helping to suppress interference from internal redundant points and local noise. Consequently, the extracted body center points provide an accurate representation of the actual spatial morphology of the standard segments, and the consistency and stability of the tower-body central axis fitting results are improved. Moreover, the consistency of the verticality inspection results obtained under different dimensional parameters of the dual-cuboid constraint frame is enhanced, thereby providing reliable support for the non-contact verticality inspection of square tower structures.
2. The proposed method was validated through field experiments conducted on a tower crane at a construction site. Four dual-cuboid constraint frame schemes with different dimensional parameters (Schemes A, B, C, and D, with dimensional errors of 20 mm, 40 mm, 60 mm, and 80 mm, respectively) were designed using the proposed method for cross-sectional point cloud slicing. The resulting tower-body verticality values were 3.48‰, 3.63‰, 3.60‰, and 3.82‰, respectively, with a mean value of 3.63‰ and a range of only 0.34‰. These results showed good agreement with the verticality values of 3.03‰ and 3.14‰ obtained using the comparative experimental Scheme E and the reference experimental Scheme F, respectively. All the results satisfied the requirement specified in GB/T 5031-2019 (*Tower Cranes*), which stipulates that the verticality shall not exceed 4‰. This finding indicates that the verticality of the tested tower crane complied with the current construction safety inspection standard and that the structure exhibited satisfactory structural stability and operational safety.
3. Field experimental validation of the proposed method was conducted using a tower crane at a construction site. In Scheme A, Scheme B, Scheme C, and Scheme D, four sets of dimensional parameters were assigned to the dual-cuboid constraint frames used for point cloud cross-sectional slicing, yielding tower-crane verticality values of 3.48‰, 3.63‰, 3.60‰, and 3.82‰, respectively. These results were reasonably close to the verticality values of 3.03‰ and 3.14‰ obtained using Scheme E and Scheme F, respectively, and showed reasonable proximity to the result of 2.82‰ obtained using Scheme G. The results indicate that the verticality of the tested tower crane complied with the applicable construction safety inspection standard and that the tower crane exhibited satisfactory structural stability and operational safety.
4. The proposed verticality inspection method takes the standard tower body structure as the research object and verifies its effectiveness and feasibility in structural axis extraction and verticality characterization.

This paper focuses on square tower structures, and the selected tower crane standard segments possess regular square cross-sectional characteristics by design. Therefore, the dual-cuboid constraint frame is adopted to perform spatial filtering of the standard segment point cloud and extract the shell point cloud, ensuring that the extracted results match the actual geometric morphology of the structure. It should also be noted that local deformation, member displacement, or point cloud occlusion may occur under long-term service conditions, applied loads, and environmental effects, causing the actual external contour of a structure to deviate from an ideal regular square. The proposed method retains a certain degree of adaptability under such conditions. In future studies, adaptive constraint frames, polygonal constraints, or surface-fitting strategies may be introduced to improve the representation of complex external structural contours and enhance geometric adaptability. Because construction at the experimental site had already been completed and the corresponding tower crane had been dismantled, supplementary tower-body verticality measurements obtained using conventional surveying instruments, such as total stations or theodolites, could not be acquired for cross-validation. Therefore, the proposed method was primarily validated through comparative analysis among different point cloud processing schemes, together with reference experiments based on the methods reported in References [30,61]. In future work, more comprehensive reference experiments will be designed by incorporating conventional measurements acquired using total stations, theodolites, and other surveying instruments to further evaluate the generalizability, accuracy, and engineering applicability of the proposed method. Differences among the verticality results obtained under different constraint schemes may arise not only from the algorithmic processing procedures but also from scanning errors, residual point cloud registration errors, and the selection of constraint-frame dimensional parameters. Future work will therefore focus on establishing an error propagation model that comprehensively incorporates instrument measurement errors, multi-station registration uncertainty, and algorithmic parameter sensitivity. This model will enable a more rigorous quantitative assessment of the confidence intervals associated with the verticality inspection results and will further strengthen the reliability evaluation of the proposed method in practical engineering applications. Furthermore, only four manually selected dimensional error parameters were investigated in the present study, and a systematic evaluation covering a broader range of constraint-frame dimensional parameters has not yet been conducted. Although the current results indicate that the verticality inspection results remained generally consistent within the tested parameter range, the optimal parameter selection and its interactions with different structural geometries and scanning conditions require further investigation. Future research will conduct a comprehensive sensitivity analysis using finer parameter intervals and a wider parameter range to establish quantitative criteria for selecting constraint-frame dimensional parameters under different engineering conditions. Such an analysis will enhance the practical applicability of the proposed method and provide clearer guidance for parameter configuration in field inspections. A quantitative evaluation framework will also be developed to assess the point reduction ratio and geometric fidelity after dual-cuboid constraint filtering, thereby more comprehensively characterizing the balance between noise suppression and geometric preservation. Although the proposed method achieved satisfactory performance in tower-crane verticality inspection, several limitations remain. First, the experimental validation was based on point cloud data acquired from only one structural type and one field site, and systematic validation has not yet been conducted for other types of square tower structures, such as communication towers and power transmission towers, or under different scanning conditions. Second, the current method is applicable only to square tower structures with rectangular cross-sections and has not been validated for non-square structures, such as towers with circular cross-sections or lattice tower structures. When the scanning distance is excessive, the point cloud density is insufficient, or severe occlusion occurs, the completeness of the shell point cloud may be inadequate, thereby affecting the accuracy of body center point extraction and central axis fitting. Consequently, the generalization of the conclusions presented in this study should be restricted to square tower structures with regular rectangular cross-sections assembled from stacked standard segments. The proposed method can be further extended to more types of tall structures, such as steel tower structures and complex truss structures, through comparative experiments under different structural forms, point cloud densities, and working conditions, so as to systematically verify the stability and applicability of the method. The current method was specifically designed for square tower structures with regular rectangular cross-sections and has not yet been validated for cylindrical towers, irregular structures, or other engineering components. Future work will investigate the extension of the constraint framework from a dual-cuboid configuration to dual-cylinder or generalized convex-hull constraints, thereby enabling its application to tower structures with circular cross-sections and more complex geometric configurations and further broadening its engineering applicability. It should be emphasized that the conclusion regarding the robustness of the proposed method to variations in dimensional parameters is currently supported by only a single field case. Validation involving a wider range of tower structures and scanning conditions will therefore be conducted in future studies to provide stronger support for this conclusion. In addition, combined with periodic LiDAR scanning and repeated observations, the dynamic time-series tracking of the verticality state of square tower structures and long-term safety monitoring can be further realized. Future research will focus on the evolution of body center points under multi-temporal point cloud conditions, the characteristics of central axis evolution, and the long-term trends of verticality parameters, aiming to further enhance the engineering application value of the proposed method in structural health monitoring.

## Figures and Tables

**Table 1:** Tower verticality inspection results.

Indicator	Scheme A	Scheme B	Scheme C	Scheme D	Scheme E	Scheme F	Scheme G
Number of contour points	6	6	6	6	6	7	7
Points for axis fitting	6	6	6	6	6	7	7
Unit direction vector	[−0.0009696 0.0033383 0.9999940]	[0.0009303 −0.0038244 −0.9999923]	[0.0007580 −0.0035152 −0.9999935]	[0.0006371 −0.0037640 −0.9999927]	[−0.0027652 −0.0012371 −0.9999954]	[−0.0029893 0.00095070 −0.9999951]	[−0.0026652 0.0010419 −0.9999959]
Azimuth angle	90°03′20″	89°56′48″	89°57′24″	89°57′49″	90°09′30″	90°10′17″	90°09′10″
Inclination angle	0°11′57″	0°13′32″	0°12′22″	0°13′07″	0°10′25″	0°10′48″	0°09′50″
Verticality	3.48‰	3.63‰	3.60‰	3.82‰	3.03‰	3.14‰	2.82‰

**Table 2:** Accuracy analysis of tower verticality inspection results.

Indicator		Scheme A	Scheme B	Scheme C	Scheme D
the verticality of the tower body and its average value		3.48‰	3.63‰	3.60‰	3.82‰
		3.63‰			
verticality reference value	**Scheme E**	3.03‰			
	**Scheme F**	3.14‰			
	**Scheme G**	2.82‰			
the relative errors corresponding to each scheme	**Scheme E**	14.85%	19.80%	18.81%	26.07%
	**Scheme F**	10.82%	15.61%	14.65%	21.66%
	**Scheme G**	23.40%	28.72%	27.65%	35.46%
the relative errors corresponding to the values in each scheme	**Scheme E**	19.80%			
	**Scheme F**	15.61%			
	**Scheme G**	28.72%			
the verticality threshold requirement		≤4‰			

## Data Availability

The data presented in this study are available in the article. Further inquiries can be directed to the corresponding authors.
